# Lung aerosol particle emission increases with age at rest and during exercise

**DOI:** 10.1073/pnas.2301145120

**Published:** 2023-05-22

**Authors:** Benedikt Schumm, Stephanie Bremer, Katharina Knödlseder, Martin Schönfelder, Rainer Hain, Luisa Semmler, Elke Lorenz, Rudolf Jörres, Henning Wackerhage, Christian J. Kähler

**Affiliations:** ^a^Department of Aerospace Engineering, Institute of Fluid Mechanics and Aerodynamics, Universität der Bundeswehr München, Neubiberg 85577, Germany; ^b^Department of Sport and Health Sciences, Professorship of Exercise Biology, Technische Universität München, Munich 80809, Germany; ^c^Department of Neurology, Klinikum rechts der Isar, Technische Universität München, Munich 81675, Germany; ^d^Klinik für Herz- und Kreislauferkrankungen, Deutsches Herzzentrum München, Technische Universität München, Munich 80636, Germany; ^e^Institute and Outpatient Clinic for Occupational, Social and Environmental Medicine, Ludwig Maximilian University of Munich, Comprehensive Pneumology Center Munich, Member of the German Center for Lung Research, Munich 80336, Germany

**Keywords:** aerosol particle emission, exercise, infection risk, pathogen transmission, SARS-CoV-2

## Abstract

Airborne respiratory aerosol particle transmission of pathogens such as severe acute respiratory syndrome-coronavirus-2 (SARS-CoV-2), influenza, or rhinoviruses plays a major role in the spread of infectious diseases. The infection risk is increased during indoor exercise, as aerosol particle emission can increase by more than 100-fold from rest to maximal exercise. Earlier studies have investigated the effect of factors such as age, sex, and body mass index (BMI), but only at rest and without taking ventilation into account. Here, we report that during both rest and exercise, subjects aged 60 to 76 y emit on average more than twice as many aerosol particles per minute than subjects aged 20 to 39 y. In terms of volume, older subjects emit on average five times as much dry volume (i.e., the residue of dried aerosol particles) than younger subjects. There was no statistically significant effect of sex or BMI within the test group. Together, this suggests that aging of the lung and respiratory tract is associated with an increased generation of aerosol particles irrespective of ventilation. Our findings demonstrate that age and exercise increase aerosol particle emission. In contrast, sex or BMI only have minor effects.

## Significance

Airborne respiratory diseases are transmitted via viruses in respiratory aerosol particles. The emission of such aerosol particles can increase by more than 100-fold from rest to maximal exercise and the risk of infection can increase by more than 10-fold, respectively. This study shows that age is another important factor that affects respiratory aerosol particle emission, as subjects aged 60 to 76 y emit more than twice as many aerosol particles at rest and during exercise and five times as much aerosol volume. This suggests that aerosol particle emission increases when the respiratory system ages.

Exercise is one of the most effective interventions to prevent (1) and treat (2) a wide range of diseases. Because of its health benefits, the World Health Organization recommends a combination of endurance and resistance exercise in its 2020 guidelines on physical activity and sedentary behavior (3). However, there are also adverse effects of exercise. One such adverse effect is that exercising individuals emit more aerosol particles per minute due to an increase of both ventilation and aerosol particle concentration (4). This raises the infection risk for others if expired aerosol particles carry pathogens such as viruses or bacteria (5–7).

In sports, aerosol-mediated infections did not appear to be a major issue before the coronavirus disease 2019 (COVID-19) because few individuals with, e.g., cold or flu symptoms took part in exercise classes. This has changed during the COVID-19 pandemic, as the participation of asymptomatic, severe acute respiratory syndrome-coronavirus-2 (SARS-CoV-2)-infected individuals in indoor group exercise classes likely explains COVID-19 outbreaks during indoor exercise sessions (8, 9).

To assess the risk of aerosol-mediated infections, researchers have developed methods to measure the concentration of aerosol particles per liter of expired air or aerosol particle emission, i.e., the number of aerosol particles emitted by one individual (10–12). This work has shown that, e.g., speaking, shouting, singing, sneezing, coughing, and exercising increases the concentration of respiratory aerosol particles when compared to rest (13).

We have recently improved the method of measuring respiratory aerosol particle emission (particles/min) which is the product of ventilation (L/min) and the concentration of respiratory aerosol particles (particles/L). Methodological improvements include the filtering of inspired air to avoid that ambient aerosol particles enter the airways and distort the results, a sampling flow rate lower than the subjects resting ventilation, measurement with compensation for oscillating exhalation flow, and the parallel measurement of ventilation and the concentration of aerosol particles in the expired air. Using this improved method, we found that aerosol emission increased on average by factor 132 from rest to maximal exercise in untrained and trained women and men aged 18 to 40 y. This large increase of particle emission was due to an about 10-fold increase in both ventilation and of the concentration of aerosol particles (4). The simultaneous measurement of aerosol particle concentration and ventilation allows determining whether a change in aerosol particle emission, particularly during exercise, is due to a change of concentration or a change of ventilation or of both variables. Also, to reduce the variation of measurement, we averaged data over at least 4 min.

Other studies have identified factors that increase the concentration of respiratory aerosol particles in expired air. These factors included higher age in a cohort of subjects aged 16 to 66 y, or a higher body mass index (BMI), or a higher product of age and BMI (14, 15). However, e.g., ref. 14 only measured the concentration of aerosol particles but not the emission of aerosol particles, which is essential for risk assessment; they also did not investigate the effect of exercise, which at moderate levels might be relevant not only during sports but also during everyday life (e.g., when climbing stairs).

To address this gap in knowledge, we designed a study to answer the following research questions:

1) What is the effect of old (60 to 76 y) versus young (20 to 39 y) age, BMI, and biological sex on ventilation, the concentration of aerosol particles per liter of expired air, and the aerosol particle emission in healthy individuals at rest and during exercise?
2) How do exercise, age, BMI, and biological sex affect aerosol particle size distribution?

## Results

### Effect of Age, Sex, and BMI at Rest and during Exercise on Aerosol Particle Emission.

A total of 80 subjects performed a graded exercise test from rest to voluntary exhaustion. Aerosol particle concentration and emission differed between age and sex groups during both rest and exercise, while there were no statistically significant differences depending on BMI or body fat percentage within the test group.

At rest, the expired air of elderly subjects contained an on average 3-fold higher respiratory aerosol particle concentration than the expired air of younger subjects [young: 105 (60 to 220) particles/L, elderly: 310 (155 to 640) particles/L; *P* < 0.001]. We also found significant differences in-between elderly men and women, with 137% higher values in elderly women when compared to elderly men [elderly women: 500 (310 to 775) particles/L, elderly men: 210 (135 to 345) particles/L; *P* = 0.008]. Similarly, there were significant differences in ventilation between age groups, with a 17% higher ventilation in the younger compared to the older group (*P* = 0.01). Moreover, young men showed a 61% higher ventilation than young women, and elderly men ventilated 24% more than elderly women (*P* < 0.001, each). Overall, at rest, elderly subjects emitted 2.7-fold more respiratory aerosol particles than those of younger subjects (*P* = 0.002). On the contrary, due to the opposite differences regarding ventilation and concentration, there was no significant difference in aerosol particle emission between the sexes of the same age group.

**Fig. 1.**
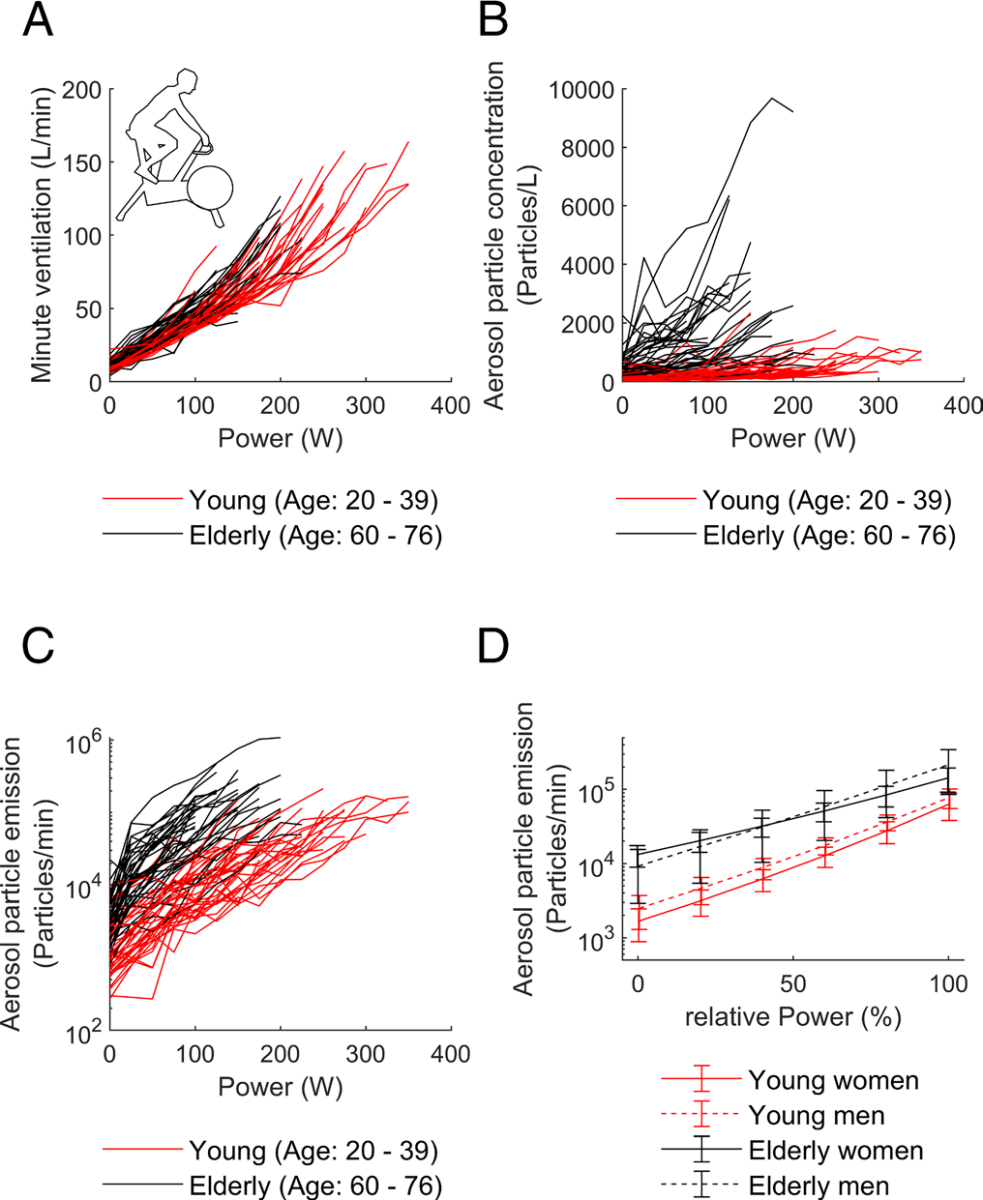
(*A*) ventilation versus power; (*B*) aerosol particle concentration versus power; (*C*) aerosol particle emission versus power; (*D*) aerosol particle emission versus relative power. (*A*–*C*) All data points from rest (power = 0 W) and for each step of the graded exercise test. The two age groups are marked by different colors illustrating the marked dependence of the aerosol particle concentration on age for both resting conditions and exercise. (*D*) Mean aerosol particle emission and 95% CI versus normalized power for each combination of age and sex. Values were derived from the individual log-linear regression fits. Relative power of 0% corresponds to resting conditions and relative power of 100% to individual maximal exercise power.

During exercise, younger subjects reached a higher maximal power output on the cycle ergometer than older subjects, and men reached higher values than women, whereby elderly men reached a higher maximal power than young women. Ventilation increased up to exhaustion in all subjects in a similar exponential manner as shown in Fig. 1*A*. The factors of increase from rest to maximal exercise were 8.6 in the young and 6.3 in the elderly subjects, whereby the young age group ventilated 52% more than the elderly subjects (young: 100 L/min, elderly: 65 L/min; *P* < 0.001). In addition, young men ventilated 53% more than young women and elderly men ventilated 57% more than elderly women (*P* < 0.001, each).

Aerosol particle concentration also increased during exercise in all subjects as shown in Fig. 1*B*. It increased 5.9-fold in the younger and 6.7-fold in the older age group. Due to the differences in the aerosol particle concentrations at rest, these increases led to different maximal aerosol particle concentrations in the two age groups, with concentrations being 3.4-fold higher in the elderly subjects than those in the young [young: 620 (390 to 940) particles/L, elderly: 2,090 (1,000 to 2,850) particles/L; *P* < 0.001].

Aerosol particle emission at maximal exercise was 2.1-fold higher in the elderly subjects compared to the younger subjects despite the lower maximal ventilation. The reason for this was the higher aerosol particle concentration [young: 54,800 (35,000 to 105,900) particles/min, elderly: 116,300 (66,300 to 196,500) particles/min; *P* = 0.003]. This corresponded to total emission increase factors from rest to maximal exercise of 49.4 in the young and 39.4 in the older subjects. There were no significant differences in aerosol particle emission between men and women. The changes in respiratory aerosol particle emission from rest to maximal exercise are shown in Fig. 1*C*.

To render the individual curves comparable, we normalized the results shown in Fig. 1*C* to the individual maximal power. To account for the exponential increase in aerosol particle emission, the individual curves were logarithmically fitted to obtain estimates of aerosol particle emission at a given percentage of maximal power. The individual estimates were then averaged for the different age and sex groups, as shown in Fig. 1*D*. As can be seen, the differences between the age groups are larger than the differences between men and women of the same age group. The average aerosol particle emission of younger subjects is reached by the older age group already on average at about 60% of their maximal workload.

**Fig. 2.**
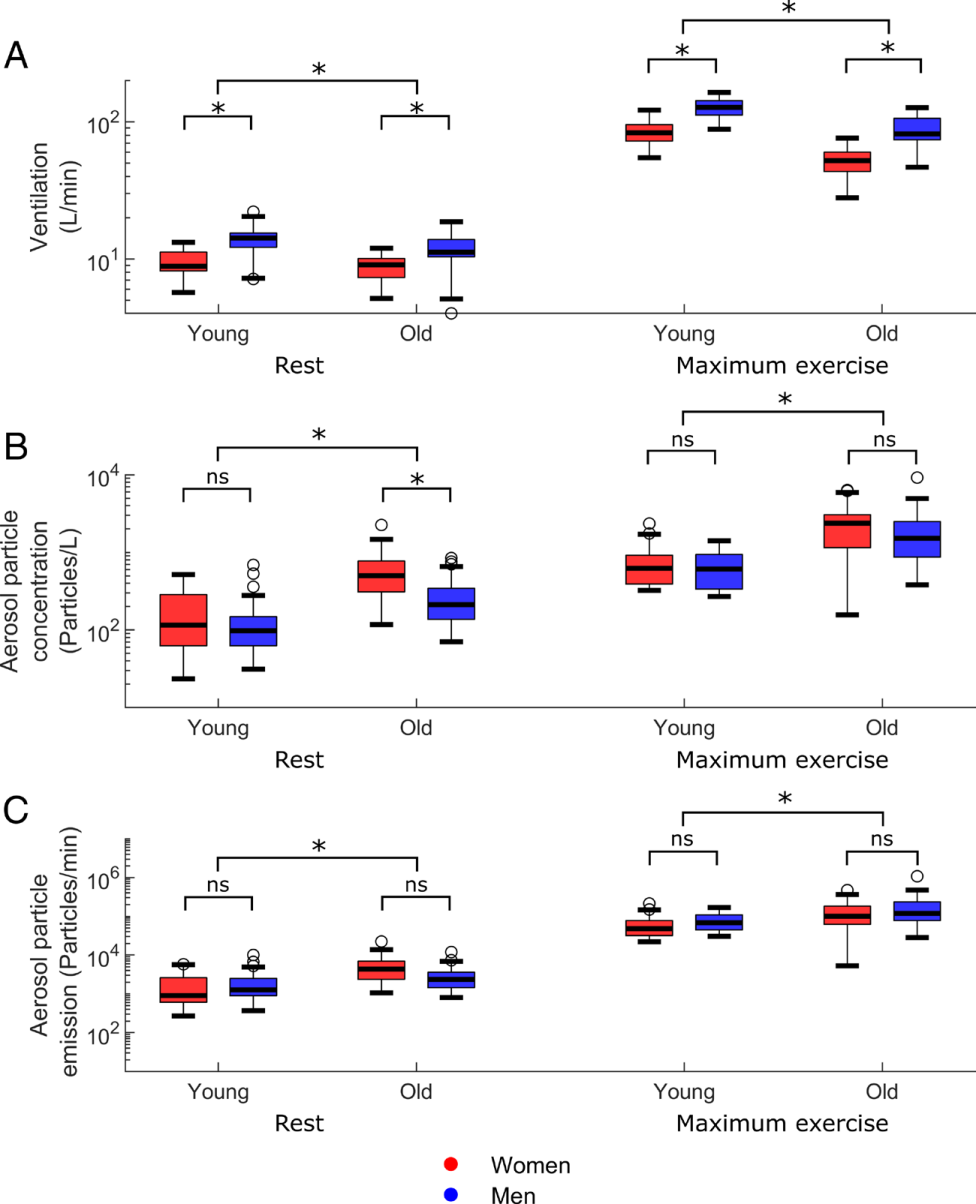
Box plots and statistical evaluation of (*A*) ventilation, (*B*) aerosol particle concentration, and (*C*) aerosol particle emission at rest (*Left* side) and at maximal exercise (*Right* side) for the two age groups and men and women. Please note the logarithmic scale on the *y* axis.

Fig. 2 visualizes ventilation, aerosol particle concentration, and aerosol particle emission for the two age groups and both sexes at rest and at maximal exercise. Significant differences between men and women of the same age group and that between the age groups are shown. In more detail, median values as well as 25th and 75th percentile of aerosol particle emission, concentration, and ventilation at rest and maximal exercise as well as maximal power are given in *SI Appendix*, Table S1 for men and women and the two age groups.

### Effect of Age on the Aerosol Particle Size Distribution and on the Volume of Aerosol Particles Emitted Per Minute.

**Fig. 3.**
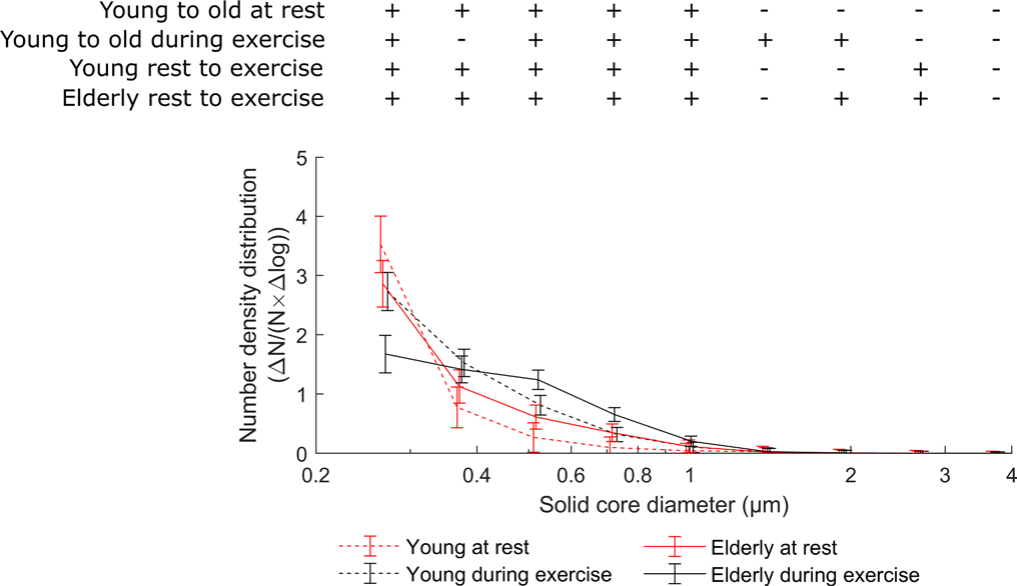
Particle size distribution at rest and during the whole exercise for the two age groups with 95% CIs. Significant differences are marked above by “+” (ANOVA with post-hoc comparisons according to Bonferroni).

In addition to aerosol particle counts, we also measured the size of the dried aerosol particle residues. Fig. 3 shows the size distribution at rest and during exercise for the two age groups. At rest, the smallest particle fraction showed high concentrations in both young and old subjects, whereas during exercise, the discrepancy between age groups became larger. Both age groups had similar relative numbers of aerosols in the particle size of 0.4 µm. However, elderly subjects had greater numbers of particles larger than 0.4 µm than those of younger subjects. The overall flattening of the distribution was more pronounced in the older subjects; thus relative to the smallest particles measured, the contribution of larger particles (0.6 to 1 µm) increased in older subjects during exercise. A statistical comparison between the particle size distributions for the two age groups at rest and during exercise showed for all bins smaller than 1.38 µm (except for one) a significant difference (*P* < 0.05, each). Comparing the size distributions between rest and exercise for the same age group, we found again significant differences for all bins up to 1.38 µm and for some of the larger bins.

The aerosol particle volume distribution can be calculated using the number frequency distribution of the dried aerosol particles when assuming spherical particles. Based on the measured distributions and emissions, the solid emission volume was calculated for different exercise intensities. To describe various conditions in sports and daily life, the results of the two age groups are illustrated for resting ventilation, maximal exercise, and exercise at 50% of individual maximal exercise intensity in Table 1. On average, older subjects emitted 3.7 to 5.8 times more dry volume than young subjects. Even during maximal exercise, young subjects emitted less aerosol particle volume than older subjects at 50% of their maximal exercise intensity.

**Table 1.** Total dry volume emission at rest, 50% of the maximal exercise intensity, and at maximal exercise for the two age groups

	Young	Elderly
	µm^3^/min	µm^3^/min
Rest	37 22–86	220 130–420
50% of maximal exercise	1,010 590–1,510	4,625 3,260–7,940
Maximal exercise	4,630 2,950–8,940	17,070 9,730–28,820

## Discussion

The main finding of this study is that subjects aged 60 to 76 y emit roughly twice as many aerosol particles than subjects aged 20 to 39 y at rest and during exercise. The greater aerosol particle emission in the elderly subjects was primarily caused by a threefold higher aerosol particle concentration in the exhaled air, when compared to younger subjects, which overcompensates the lower ventilation. These data suggest that older exercisers should protect themselves and others more against aerosol-mediated infections especially during indoor group exercise classes. This is especially important for other elderly exercisers because the risk of such infections is higher in elderly subjects and because elderly typically suffer from higher morbidity and mortality when infected with aerosol-transmitted pathogens such as SARS-CoV-2 or influenza. During indoor group exercise sessions, the risk of infection increases due to the high emission of aerosol particles by an exerciser. Also, the simultaneously very high inhalation rate of uninfected exercisers increases their risk of infection. A recent infection risk simulation using aerosol particle emission data from real-life endurance and resistance exercise estimated that the risk of infection is by up to 2.5-fold higher in exercisers compared to nonexercisers (16).

Elderly subjects already reached on average the same aerosol particle emission at 60% of maximal exercise intensity that younger individuals reached at maximal exercise. While age had a large effect on aerosol particle emission, the effect of sex on aerosol particle emission was small and there was no significant effect of BMI or body fat percentage, in contrast to earlier studies (14, 17). The results demonstrate that age is a major determinant of aerosol particle emission from the lung. People of older age may reach emission levels of exhaled aerosol particles at moderate exercise encountered in daily life that younger subjects reach only during intensive or maximal exercise.

Several studies have measured aerosol particle concentrations in exhaled air at rest or during exercise (11, 13, 14, 18), but ventilation was not measured in these studies. This limits the interpretation of this data because ventilation needs to be measured to be able to calculate the emission of aerosol particles per unit of time by an individual and to calculate the concentration of aerosol particles in the room air for infection risk estimations. This is particularly true if subjects of different age, sex, and exercise capacity are investigated during exercise, where ventilation can change by 10-fold or more. Moreover, subjects differ in their ventilation at a given workload.

There are several mechanisms of respiratory aerosol particle generation within the upper or lower airways. A key mechanism is the mechanically induced rupture of liquid films, which occurs within the human lung during collapse–reopening cycles of the small airways (11, 19–21). The underlying mechanisms of the aerosol particle concentration and emission increase observed by us are probably due to differences in the function of the peripheral airways and the cycle of collapse and reopening (11, 19–21). This cycle is linked to the periodic rupture of the fluid film covering the mucosa, leading to the generation of aerosols (19). This is difficult to investigate, however, as it would involve visualizing and quantifying aerosol particle generation within the human airways.

Respiratory aerosol particle generation might also depend on properties of the inhaled air; this has been shown for ambient air humidity and the inhalation of saline-containing water droplets (15), which may alter the surface tension of the liquid film in the lung and thereby the generation of aerosol particles. In our study, it is unlikely that this played a role, as we prevented the inhalation of ambient air aerosol particles and kept ambient air conditions as constant as possible. The relative humidity varied, but this was the same in older and younger subjects and markedly reduced if expressed as absolute humidity after considering temperature. Peripheral airway morphology and function changes with increasing age even in healthy subjects (22, 23) and this is likely to underlie the observed differences between age groups. However, a detailed mechanistic understanding would probably require interventions targeting the function of the peripheral airways. Our study aimed to quantify the sensitivity of the particle emission on parameters such as exercise level and age to provide data for practical purposes of risk prediction relying on easy-to-assess determinants. As such major determinants, we identified age and, to a lesser extent, sex.

For predicting the risk of SARS-CoV-2, influenza, or other infections, the amount of exhaled aerosol particles carrying infectious viruses is important. Our measuring device covered a range from 200 nm to 10 µm in particle diameter. Studies in which smaller particles (> 10 nm) were detected with specific instruments have shown that a large proportion of exhaled particles can be smaller than the lower threshold value of our instrument (24). Accordingly, the peak at the smallest fraction of dried particles in our data was due to the cutoff value of size at the lower end in our assessments. The coronavirus has a size of about 100 nm (25). Therefore, in principle, the particles measured by us could have carried at least one virus, or even more before the drying process. In additin we found a shift in particle size distribution between rest and exercise and differences between age groups. It suggests that the mechanisms underlying aerosol particle generation in the lung contribute differently under these conditions. The shift in the distribution toward larger particles suggests a larger capacity for carrying exhaled virus in the elderly during exercise. To compare the potential risk under different conditions and between age groups, we calculated the cumulative dry volume of exhaled aerosol particles as a function of the emitted number and the size distribution. Due to the shifts in distribution toward larger diameters, the difference between age groups and the dependence on exercise became even greater.

In reality, the virus load of the exhaled particles depends on the virus concentration at the location of origin. This concentration could also depend on the type of the virus; for example, rhinovirus has not been detected in exhaled air (26), which is plausible given its major location. Regarding SARS-CoV-2, the location is probably dependent on disease severity. Coarse particles with a size > 5 µm contain less virus than the corresponding finer fraction (27, 28). There is no information about the size-dependent viral load of aerosol particles < 5 µm. This size range applied to 99.6% of particles in our measurements and pointed toward an origin in peripheral airways of aerosols in which the SARS-CoV-2 virus is likely to be found at least in moderate-to-severe diseases. Based on this, we computed the total volumes of aerosol particles as potential measures of maximal virus load, assuming a constant virus concentration within this size range.

Our observed average values of aerosol particle concentrations at rest, in the absence of infection, were in the range of approximately 250 particles/L, i.e., values reported previously (10, 12, 14). However, our aerosol particle emission values at rest were up to 10-fold higher than the values reported in the literature (13, 29), probably due to different measurement techniques. It has also been reported that talking or singing at medium or loud volumes can increase aerosol particle concentrations by twofold and 10-fold or up to 55-fold compared to the aerosol particle concentrations measured at resting ventilation (13, 30), which is comparable to the magnitude of increase measured during exercise (3.6-fold for resistance and 10-fold during endurance exercise) (4, 16). As previously shown (4), aerosol particle concentration and emission increase exponentially with increasing exercise intensity. The aerosol particle concentration during and at maximal exercise observed by us was in line with values reported for forced breathing maneuvers (mean: 1,300 particles/L, range: 69 to 5,300 particles/L) or partial breathing maneuvers (mean: 2,500 particles/L, range: 330 to 13,000 particles/L) in healthy subjects (12). Moreover, the factor of increase of aerosol particle emission from rest to maximal exercise ranged between 25 and 63 in our study, which is similar to previous results obtained by ref. 18, showing a 58-fold increase in total aerosol particle number at an exercise level of approximately 70% of maximal heart rate.

The aerosol particle emission values at maximal exercise observed by us can be compared with those of one study, in which also particle concentration and ventilation were measured during exercise (29). For a group of 25 subjects with an average age of 36.4 ± 14.9 y, these authors found emission rates of about 36,000 particles/min; this is comparable to our median value of about 55,000 particles/min obtained in the younger age group at a similar exercise intensity.

The discrepancies between studies highlight the important point that the methods for measuring the concentration of aerosol particles differ greatly and that ventilation is rarely measured, thus not allowing for the assessment of proper emission rates as measures of risk from exhaled air. Moreover, the measuring range of the particle counters used often differed, with most studies specifying 0.5 µm as the lower detection limit (13, 29, 30). In contrast, our measuring device measured down to approximately 0.2 µm. This is relevant because in this size range the particle number increases strongly with decreasing size (24). Also, we measured dry particle cores, which also could explain differences to other studies. In addition, differences in emission maneuvers (breathing, speaking, singing) involving different contributions from different particle generation mechanisms also play a role. As a result of all these factors, there may be large differences between studies. As a further important factor, we identified age with its marked influence on aerosol particle concentration and emission, thereby providing a simple characteristic to standardize study populations and make studies more comparable.

### Limitations.

Our study has several limitations. First, while measuring the emission of pathogens is the ultimate goal, we only measured the emission of aerosol particles and not that of pathogens. Second, our results are from healthy subjects, as we excluded subjects with respiratory diseases and acute respiratory illness. This might be relevant as aerosol particle concentrations can increase in infected individuals (14) presumably because a respiratory infection alters the properties and function of peripheral airways that determine aerosol particle production. Third, not all subjects were able of completing the graded exercise test until objective exhaustion criteria were reached. Fourth, we also did not attempt to control subjects’ fluid intake prior to the tests, which theoretically could have influenced airway mucosa. As the subjects had the opportunity to drink ad libitum before the start and the exercise test was of short duration, it is unlikely that dehydration played a role. In respect to the hydration status experiments with intravenous isotonic saline infusion in the order of 30 mL/kg of body weight, the membrane conductivity of the lung decreased by about 10 to 15% (31, 32). Similar small effects have been found for massive hypertonic saline inhalation (33). This suggests that large amounts of fluid are needed to cause measurable effects on functional parameters that are indicators of mucosal properties or dehydration. Alternatively, dehydration from inhaled air could have played a role, but the variation in absolute humidity, i.e., the water content of inhaled air, was small.

## Conclusion

In this study, we investigated respiratory aerosol particle emission at rest and during a graded exercise test to exhaustion in 80 healthy men and women aged either 20 to 39 y or 60 to 76 y, and with different BMIs. The main finding was that the elderly subjects emitted on average more than twice as many aerosol particles per minute and five times as much dry volume than those of subjects aged 20 to 39 y, whereas there was only a small difference between women and men. This is important information for planning mitigation measures especially for indoor sport facilities during infection waves or future pandemics. Whether this is linked to differences in viral load of exhaled air and the risk of transmitting infections must be clarified in further studies.

## Methods

### Study Design and Participant Characteristics.

We conducted a monocentric cohort study continuously measuring respiratory ventilation, concentration of aerosol particles in expired air, and aerosol particle emission both at rest and during a graded exercise test until exhaustion.

Subjects and staff were tested for SARS-CoV-2 with an antigen test before testing. Eligible participants were between 20 and 39 y (young adults) or 60 and 76 y (elderly) old, nonsmokers, without any respiratory diseases, and did not suffer a SARS-CoV-2 infection in the past 4 wk before enrolment. The absence of relevant clinical conditions was confirmed by taking a clinical history and standard clinical tests including resting electrocardiogram and blood pressure assessment. Height, weight, and body composition (InBody 770, InBody Co., Ltd., Eschborn, Germany) were determined prior to the tests. We recruited a total of 80 subjects stratified into four groups of 20 subjects according to age group and sex. Ambient testing conditions were: temperature: 24.8 ± 1.5 °C (20.6 °C to 28.1 °C), air pressure: 952.4 ± 5.9 hPa (941 hPa to 964 hPa), relative humidity: 24.7 ± 10.8% (10.2 to 52.1%), and absolute humidity: 19.0 ± 1.0 g/m^3^ (17.5 to 21.2 g/m^3^).

All measurements and procedures were approved by the medical ethical committee of the Technical University Munich. Prior to study enrolment, participants were informed about the risks and benefits of participating in this study and a written informed consent was obtained from all participants.

#### Study population.

The two age groups comprised 40 subjects from age 20 to 39 y and 40 subjects from age 60 to 76 y. Each age group comprised 20 women and 20 men. Their characteristics are shown in Table 2.

**Table 2.** Participant characteristics; mean values and SD as well as range

	Young (20–39 y)		Elderly (60–76 y)	
	women	men	women	men
n	20	20	20	20
Age (years)	25.2 ± 4.3	29.0 ± 4.8	68.6 ± 4.7	66.2 ± 3.8
	(20–39)	(20–37)	(60–76)	(60–73)
Height (cm)	165.4 ± 6.5	184.7 ± 4.2	165.9 ± 5.9	176.5 ± 6.9
	(151–178)	(177–193)	(158–179)	(165–192)
Weight (kg)	61.3 ± 7.4	85.8 ± 11.6	67.1 ± 11.1	80.7 ± 8.6
	(51.5–78.4)	(67.8–107.4)	(49.6–89.5)	(65.3–99.0)
BMI (kg/m^2^)	22.5 ± 3.1	25.1 ± 3.0	24.4 ± 3.8	25.9 ± 2.6
	(18.0–29.3)	(20.6–30.9)	(18.5–31.5)	(22.4–33.3)
Body fat (%)	24.3 ± 6.7	16.2 ± 7.0	31.0 ± 7.6	22.1 ± 6.0
	(11.6–37.5)	(7.7–30.0)	(16.0–44.1)	(8.4–31.1)

Height was significantly dependent on sex in both age groups, whereby only in males, there was an additional dependence on age (*P* < 0.01 each). Weight was dependent on sex but not on age (*P* < 0.001). The same was true for BMI (*P* < 0.05). Body fat percentage increased with age and was higher in women compared to men (*P* < 0.001 each).

### Cardiopulmonary Exercise Test.

Participants performed a cardiopulmonary exercise test on a bicycle ergometer (Ergoline Ergoselect 200, Lode, Groningen, Netherlands). After a 5-min resting phase on the bicycle ergometer, the subjects were encouraged to follow a graded exercise test protocol starting at 25 W (elderly) or 50 W (young adults), with 25 W increments every 4 min until exhaustion. After each 25 W increment, the participants were asked to indicate their rate of perceived exertion (6 to 20; Borg Perception, Hasselby, Sweden) using their hands only. The subjects were instructed not to speak throughout the test.

Ventilation, oxygen consumption, carbon dioxide production, and other related variables were assessed via breath-by-breath analysis using a stationary cardiopulmonary exercise testing system (Metalyzer; Cortex Medical^TM^, Leipzig, Germany). The peak oxygen uptake was calculated at the highest VO_2_ levels. Besides the recording of ventilation parameters, aerosol particle concentrations both at rest and during exercise were monitored.

Out of the 80 participants, 37 met the objective exhaustion criteria of a respiratory exchange ratio (RER) > 1.0 (64 subjects with an RER > 0.95) or 73 reached a heart rate > 85% of the predicted maximal. Two elderly participants had to terminate the exercise test due to high blood pressure occurring at the end of the test (systolic blood pressure > 220 mmHg). All the tested subjects were evaluated.

### Aerosol Particle Measurement.

Respiratory particle concentration was measured using an optical particle counter (Promo 3,000 particle spectrometer combined with a Welas 2300 sensor; Palas GmbH, Karlsruhe, Germany) in parallel to the ventilation measurement. A constant flow of 5 L/min was sampled from the expired air stream behind the spirometry sensor and valve system. All subjects ventilated more than 5 L/min even at rest so that sampling was always possible without the need for adding air that was not coming from the subject. To avoid a bias from inhaled ambient aerosol particles, the inspired air was filtered by a H14 filter that was driven by a ventilator and supplied in excess without pressure to avoid any impairment of inhalation during exercise. Also, to shield the measurement line from spurious aerosol particles from ambient air, exercise tests were performed in a clean air tent with a mobile room air cleaner (TAC V+ GR/BKII, Trotec, Heinsberg, Germany), again equipped with an internal H14 filter, filtering and circulating the air inside the tent. To prevent accumulation of CO_2_, the expired air was removed from the tent, while a second mobile room air cleaner (E15, Trotec, Heinsberg, Germany) flushed filtered air into it.

The complete system starting at the two-way valve and the flow sensor up to the particle spectrometer sensor head was heated to at least 40°C to avoid condensation and associated aerosol particle losses on the surfaces and to measure dry particles.

Using this equipment, respiratory particle concentration was monitored continuously throughout the whole test from rest until exhaustion. Data were averaged for each step of the exercise test, i.e., 5 min for resting ventilation, 4 min for each completed intensity step, or at least 60 s in the last exercise intensity step; if this included less than 60 s, the last completed step was taken for analysis.

In addition to particle counts, the spectrometer measured particle size distribution via the refractive index of latex particles as usual (user manual, Palas, Heinsberg, Germany). To prevent larger particles from settling inside the measurement system, the tubing was kept as short as possible ( ≈50 cm). However, we cannot completely exclude separation of larger particles at the flaps of the two-way valve due to its 90° deflection angle.

### Data Processing and Statistical Analyses.

All data were extracted from the raw files and processed with Matlab (R2021b, The MathWorks Inc., Natick, MA, USA). Mean values were calculated for each time interval. The analysis of the data showed that only the ventilation data of the groups were normally distributed, but not the aerosol particle concentration or emission. In the presence of normal distribution, the median and the mean values were similar. Therefore, for uniformity, the median and the values of the 25th and 75th percentiles were given for all data. Statistical comparisons between groups were performed by one-way or two-way ANOVA models including interaction terms if necessary, with the Bonferroni post-hoc test and the Levene test for variance homogeneity. In addition, linear regression analysis was used to describe the relationship between power and logarithmically transformed values of aerosol particle emission via intercept and slope for each individual. Significance was assumed for *P* < 0.05. All statistical analyses were performed with the package SPSS (IBM, Armonk, NY, USA).

## Supplementary Material

Appendix 01 (PDF)

Dataset S01 (PDF)

## Data Availability

All study data are included in the article and/or *SI Appendix*.
