# Supplementary material for: Lung aerosol particle emission increases with age at rest and during exercise
Source: Proc Natl Acad Sci U S A. 2023 May 22;120(22):e2301145120. doi: 10.1073/pnas.2301145120 (PMC10235964; doi:10.1073/pnas.2301145120)
Supplement: Supplementary file 1 — Appendix 01 (PDF) [file pnas.2301145120.sapp.pdf]

Supporting Information for

Lung aerosol particle emission increases with age at rest and during exercise

Benedikt Schumm, Stephanie Bremer, Katharina Knödlseder, Martin Schönfelder, Rainer Hain, Luisa Semmler, Elke Lorenz, Rudolf Jörres, Henning Wackerhage* and Christian J. Kähler*

Corresponding Author: Benedikt Schumm,
benedikt.schumm@unibw.de

This PDF file includes:

Table S1

Other supporting materials for this manuscript include the following:

Dataset

Table S1: Median and values of the 25th and 75th percentile for aerosol particle emission, aerosol particle concentration, and ventilation at rest and at maximum exercise together with maximum power and $VO_{2,\,max}$ for each participant group. P-Values are given indicating differences between men and women of the same age group and between the age groups, all men and women as well as the interaction term

		Young		Elderly	
		women	men	women	men
At rest	Aerosol particle emission (Particles/min)	910<br>(610 – 2630)	1,270<br>(900 – 2,520)	4,370<br>(2,380 – 6,980)	2,370<br>(1,460 – 3,630)
		$p > 0.05$		$p > 0.05$	
		Age group:	$p = 0.002$		
		Sex:	$p > 0.05$		
		Interaction:	$p = 0.05$		
	Aerosol particle concentration (Particles/L)	115<br>(62 – 286)	100<br>(62 – 150)	500<br>(310 – 775)	210<br>(140 – 340)
		$p > 0.05$		$p = 0.009$	
		Age group:	$p = 0.01$		
		Sex:	$p < 0.001$		
		Interaction:	$p > 0.05$		
	Ventilation (L/min)	8.8<br>(8.2 – 11.2)	14.2<br>(12.2 – 15.5)	9.1<br>(7.3 – 10.1)	11.2<br>(10.4 – 13.9)
		$p < 0.001$		$p < 0.001$	
		Age group:	$p < 0.001$		
		Sex:	$p = 0.008$		
		Interaction:	$p = 0.021$		

At maximum exercise	Power (W)	175<br>(150 – 187.5)	262.5<br>(237.5 – 300)	100<br>(100 – 125)	175<br>(150 – 200)
		$p < 0.001$		$p < 0.001$	
		Age group:	$p < 0.001$		
		Sex:	$p < 0.001$		
		Interaction:	$p > 0.05$		
	$VO_{2,\,max}$ (mL/(min kg))	43.0<br>(39.0 – 49.0)	47.0<br>(41.0 – 55.0)	28.0<br>(24.5 – 31.5)	35.5<br>(33.0 – 38.5)
		$p > 0.05$		$p < 0.001$	
		Age group:	$p < 0.001$		
		Sex:	$p < 0.001$		
		Interaction:	$p > 0.05$		
	Aerosol particle emission (Particles/min)	48,100<br>(31,900 – 77,800)	68,400<br>(44,700 – 109,500)	100,700<br>(62,500 – 182,800)	119,400<br>(77,800 – 236,400)
		$p > 0.05$		$p > 0.05$	
		Age group:	$p = 0.003$		
		Sex:	$p > 0.05$		
		Interaction:	$p > 0.05$		
	Aerosol particle concentration (Particles/L)	620<br>(410 – 920)	610<br>(340 – 940)	2,380<br>(1,150 – 3,050)	1,510<br>(870 – 2,500)
		$p > 0.05$		$p > 0.05$	
		Age group:	$p < 0.001$		
		Sex:	$p > 0.05$		
		Interaction:	$p > 0.05$		
	Ventilation (L/min)	83.1<br>(72.3 – 95.3)	127.2<br>(112.1 – 142.8)	52.2<br>(43.6 – 60.1)	81.7<br>(74.0 – 105.9)
		$p < 0.001$		$p < 0.001$	
		Age group:	$p < 0.001$		
		Sex:	$p < 0.001$		
		Interaction:	$p > 0.05$		
