# Supplementary material for: Lung aerosol particle emission increases with age at rest and during exercise
Source: Proc Natl Acad Sci U S A. 2023 May 22;120(22):e2301145120. doi: 10.1073/pnas.2301145120 (PMC10235964; doi:10.1073/pnas.2301145120)
Supplement: Supplementary file 2 — Dataset S01 (PDF) [file pnas.2301145120.sd01.pdf]

Ventilation in L/min

Old	Subject	1=Women; 2=Men	Power in W													
			0	25	50	75	100	125	150	175	200	225	250	275	300	325
	1	2	10.7	17.8	23.4	29.7	35.8	46.9	61.7	77.8	NA	NA	NA	NA	NA	NA
	2	2	14.2	22.8	27.1	32.1	45.4	56.8	81.1	NA	NA	NA	NA	NA	NA	NA
	3	1	7.4	16.1	26.1	38.3	45.9	NA	NA	NA	NA	NA	NA	NA	NA	NA
	4	2	4.0	13.4	15.4	19.9	42.3	45.6	46.7	NA	NA	NA	NA	NA	NA	NA
	5	2	13.8	23.3	30.7	37.0	40.3	50.5	59.7	71.0	86.5	96.9	NA	NA	NA	NA
	6	2	11.3	23.7	30.0	37.5	48.6	62.5	83.1	103.8	NA	NA	NA	NA	NA	NA
	7	1	12.0	19.1	28.5	41.7	58.1	76.1	NA	NA	NA	NA	NA	NA	NA	NA
	8	1	9.1	18.2	29.1	36.7	NA	NA	NA	NA	NA	NA	NA	NA	NA	NA
	9	1	7.9	15.8	24.0	33.0	49.8	67.5	NA	NA	NA	NA	NA	NA	NA	NA
	10	1	9.0	18.6	26.8	33.7	NA	NA	NA	NA	NA	NA	NA	NA	NA	NA
	11	1	11.6	24.6	31.1	42.3	50.5	NA	NA	NA	NA	NA	NA	NA	NA	NA
	12	2	14.0	17.5	23.8	32.5	41.5	50.9	66.1	73.3	NA	NA	NA	NA	NA	NA
	13	1	5.5	16.3	32.5	44.0	53.8	NA	NA	NA	NA	NA	NA	NA	NA	NA
	14	2	11.4	20.3	25.8	34.8	43.7	55.6	69.4	89.8	112.9	NA	NA	NA	NA	NA
	15	1	10.2	22.5	31.7	39.6	49.7	60.4	NA	NA	NA	NA	NA	NA	NA	NA
	16	2	18.7	34.2	38.6	52.2	59.9	74.4	81.6	102.5	126.7	NA	NA	NA	NA	NA
	17	1	9.5	21.7	37.0	53.5	60.6	NA	NA	NA	NA	NA	NA	NA	NA	NA
	18	1	9.4	16.7	22.0	42.3	59.2	NA	NA	NA	NA	NA	NA	NA	NA	NA
	19	2	15.4	25.9	35.9	44.9	48.1	62.5	72.5	87.2	105.4	NA	NA	NA	NA	NA
	20	1	9.9	8.2	22.6	27.9	NA	NA	NA	NA	NA	NA	NA	NA	NA	NA
	21	1	10.8	19.1	26.5	34.2	42.4	49.9	59.7	NA	NA	NA	NA	NA	NA	NA
	22	2	10.3	17.7	23.8	30.5	37.8	50.1	67.9	95.1	NA	NA	NA	NA	NA	NA
	23	2	11.1	28.5	38.3	46.4	53.2	62.8	74.3	92.8	108.2	NA	NA	NA	NA	NA
	24	1	7.0	14.7	24.9	40.5	NA	NA	NA	NA	NA	NA	NA	NA	NA	NA
	25	2	10.2	21.5	26.4	31.1	39.7	47.7	61.3	73.7	NA	NA	NA	NA	NA	NA
	26	1	9.3	18.2	26.6	19.2	47.2	NA	NA	NA	NA	NA	NA	NA	NA	NA
	27	2	13.1	22.7	28.6	37.0	47.2	62.9	79.5	NA	NA	NA	NA	NA	NA	NA
	28	2	10.9	17.9	22.8	28.0	37.8	46.2	55.5	67.1	73.7	74.0	NA	NA	NA	NA
	29	1	5.2	16.8	26.3	34.6	44.5	56.6	NA	NA	NA	NA	NA	NA	NA	NA
	30	2	10.4	19.9	25.8	33.2	40.5	50.0	60.7	74.0	NA	NA	NA	NA	NA	NA
	31	1	5.8	18.6	26.2	34.8	48.9	NA	NA	NA	NA	NA	NA	NA	NA	NA
	32	1	7.9	11.4	20.5	33.7	43.9	38.1	41.1	NA	NA	NA	NA	NA	NA	NA
	33	2	14.7	31.2	38.2	44.7	55.2	65.9	82.3	NA	NA	NA	NA	NA	NA	NA
	34	2	10.5	25.5	36.9	45.3	56.3	68.2	85.9	106.0	117.3	NA	NA	NA	NA	NA
	35	1	11.1	20.5	31.5	45.1	59.0	NA	NA	NA	NA	NA	NA	NA	NA	NA
	36	2	11.7	23.3	32.9	38.1	46.6	59.0	NA	NA	NA	NA	NA	NA	NA	NA
	37	2	7.1	24.3	30.4	39.5	47.7	56.4	71.3	85.5	106.4	NA	NA	NA	NA	NA
	38	1	7.9	17.9	28.6	40.0	55.9	62.4	NA	NA	NA	NA	NA	NA	NA	NA
	39	2	8.3	17.7	26.3	30.7	37.3	66.1	74.8	NA	NA	NA	NA	NA	NA	NA
	40	1	7.3	22.5	36.8	41.0	50.5	NA	NA	NA	NA	NA	NA	NA	NA	NA

Young	Subject	1=Women; 2=Men	Power in W													
			0	50	75	100	125	150	175	200	225	250	275	300	325	350
	1	2	16.9	26.2	34.3	40.9	48.4	53.7	60.2	68.4	76.9	86.0	97.0	106.4	122.2	134.7
	2	2	13.8	28.8	37.5	46.4	55.4	64.8	78.2	91.5	111.7	136.1	157.5	NA	NA	NA
	3	2	11.8	23.9	30.2	36.2	43.4	50.4	58.6	70.5	84.7	103.8	NA	NA	NA	NA
	4	2	7.2	20.2	26.2	34.8	42.7	55.9	73.3	88.3	NA	NA	NA	NA	NA	NA
	5	2	11.6	24.5	39.3	50.4	61.5	70.1	88.5	NA	NA	NA	NA	NA	NA	NA
	6	2	13.2	22.4	33.6	44.5	55.8	79.3	102.1	NA	NA	NA	NA	NA	NA	NA
	7	1	15.0	30.8	37.3	42.8	49.8	58.4	53.7	51.9	80.4	94.8	105.3	NA	NA	NA
	8	2	10.4	21.8	28.7	36.1	43.7	52.6	63.3	NA	NA	NA	NA	NA	NA	NA
	9	1	9.4	24.0	30.4	36.9	40.7	50.8	60.5	74.0	92.0	NA	NA	NA	NA	NA
	10	2	12.7	27.8	37.3	48.3	62.0	84.3	NA	NA	NA	NA	NA	NA	NA	NA
	11	1	15.9	32.2	39.6	45.9	52.3	59.1	62.5	68.1	86.1	98.6	119.6	144.8	148.6	NA
	12	2	14.6	25.0	29.1	36.5	44.6	51.1	55.1	65.7	74.7	86.1	97.3	112.6	130.0	135.1
	13	2	11.4	34.5	51.6	75.6	92.6	NA	NA	NA	NA	NA	NA	NA	NA	NA
	14	1	11.2	24.4	35.2	44.9	58.5	77.6	NA	NA	NA	NA	NA	NA	NA	NA
	15	1	6.8	21.7	30.7	36.2	41.3	52.0	59.3	75.5	98.6	NA	NA	NA	NA	NA
	16	1	14.6	25.3	34.0	40.7	44.8	53.1	61.0	67.0	70.7	75.7	87.8	118.9	NA	NA
	17	2	12.5	26.0	34.5	45.8	56.8	69.7	88.3	NA	NA	NA	NA	NA	NA	NA
	18	1	12.6	26.5	33.4	39.6	46.0	53.0	66.7	73.8	86.9	103.2	122.1	NA	NA	NA
	19	2	10.3	22.0	31.7	43.0	58.2	76.8	98.6	NA	NA	NA	NA	NA	NA	NA
	20	1	13.6	22.5	33.4	42.5	51.1	65.3	75.1	106.0	121.1	NA	NA	NA	NA	NA
	21	2	9.5	22.1	33.7	42.9	54.7	NA	NA	NA	NA	NA	NA	NA	NA	NA
	22	1	11.2	24.0	36.7	48.0	59.9	76.0	NA	NA	NA	NA	NA	NA	NA	NA
	23	1	13.1	23.0	30.1	37.1	45.2	53.4	65.7	85.1	109.0	134.7	NA	NA	NA	NA
	24	2	14.8	24.5	29.6	37.2	46.6	56.5	63.2	73.7	96.4	119.7	NA	NA	NA	NA
	25	2	17.0	29.0	36.2	43.7	49.4	57.7	65.9	75.4	87.2	93.5	130.3	149.3	NA	NA
	26	2	13.1	23.5	31.2	37.7	45.6	50.5	55.3	63.8	73.4	85.8	98.5	115.1	137.0	163.9
	27	2	8.0	18.0	27.8	34.4	44.0	53.7	68.7	NA	NA	NA	NA	NA	NA	NA
	28	1	17.3	24.1	35.3	42.9	51.1	59.8	69.9	96.3	110.3	131.8	NA	NA	NA	NA
	29	2	14.6	28.6	36.9	43.3	52.5	62.0	71.3	88.0	120.7	147.2	NA	NA	NA	NA
	30	2	8.4	19.7	30.5	39.1	51.8	67.6	81.8	NA	NA	NA	NA	NA	NA	NA
	31	1	8.6	21.7	29.4	36.9	43.7	52.9	67.3	85.1	NA	NA	NA	NA	NA	NA
	32	1	22.1	25.9	35.0	48.1	56.2	70.3	86.5	112.4	138.5	NA	NA	NA	NA	NA
	33	2	10.4	19.3	29.3	40.8	48.2	55.8	66.2	75.4	86.3	101.0	122.5	NA	NA	NA
	34	2	7.4	21.9	28.4	34.7	41.7	49.6	59.9	76.8	102.9	121.7	NA	NA	NA	NA
	35	1	8.8	20.4	27.6	37.0	46.0	63.8	NA	NA	NA	NA	NA	NA	NA	NA
	36	1	8.8	25.1	33.0	41.9	48.4	57.7	70.6	93.5	NA	NA	NA	NA	NA	NA
	37	1	7.2	25.3	36.1	48.0	63.2	81.8	NA	NA	NA	NA	NA	NA	NA	NA
	38	1	8.9	26.0	42.0	62.2	NA	NA	NA	NA	NA	NA	NA	NA	NA	NA
	39	1	5.7	19.5	29.3	38.0	54.7	81.3	NA	NA	NA	NA	NA	NA	NA	NA
	40	1	8.3	21.6	31.1	40.6	48.0	59.3	74.9	97.2	NA	NA	NA	NA	NA	NA

Aerosol particle concentration in Particles/L

		1=Women;	Power in W												
**Old**	Subject	2=Men	0	25	50	75	100	125	150	175	200	225	250	275	300	325
	1	2	266.46	283.96	411.37	646.39	646.59	861.93	1,537.51	1,537.43	NA	NA	NA	NA	NA	NA
	2	2	846.66	1,332.17	1,440.02	1,429.93	2,301.28	2,731.85	4,758.90	NA	NA	NA	NA	NA	NA	NA
	3	1	658.68	1,097.28	1,224.55	1,380.93	1,312.59	NA	NA	NA	NA	NA	NA	NA	NA	NA
	4	2	760.74	911.64	1,166.40	1,205.36	1,244.25	1,783.21	2,293.11	NA	NA	NA	NA	NA	NA	NA
	5	2	290.00	489.91	489.75	450.81	646.77	431.24	656.30	548.67	568.35	510.19	NA	NA	NA	NA
	6	2	235.11	538.58	538.40	744.14	900.60	1,409.86	1,968.06	2,408.75	NA	NA	NA	NA	NA	NA
	7	1	415.52	1,096.89	1,400.28	2,301.24	4,111.79	6,217.60	NA	NA	NA	NA	NA	NA	NA	NA
	8	1	924.43	1,351.36	1,507.80	2,066.44	NA	NA	NA	NA	NA	NA	NA	NA	NA	NA
	9	1	305.76	646.54	646.53	1,762.83	3,261.38	2,806.55	NA	NA	NA	NA	NA	NA	NA	NA
	10	1	117.60	117.59	215.47	156.73	NA	NA	NA	NA	NA	NA	NA	NA	NA	NA
	11	1	141.06	1,919.03	2,026.83	2,888.36	2,570.68	NA	NA	NA	NA	NA	NA	NA	NA	NA
	12	2	164.64	166.46	156.80	107.78	293.88	489.67	577.78	642.62	NA	NA	NA	NA	NA	NA
	13	1	313.69	342.80	372.05	460.25	766.01	NA	NA	NA	NA	NA	NA	NA	NA	NA
	14	2	70.57	146.95	205.73	313.44	235.15	303.65	538.68	685.60	950.03	NA	NA	NA	NA	NA
	15	1	736.77	1,233.62	2,114.77	2,466.73	2,967.35	3,368.79	NA	NA	NA	NA	NA	NA	NA	NA
	16	2	117.61	225.09	401.57	528.92	655.94	1,057.46	1,870.01	2,340.17	2,593.15	NA	NA	NA	NA	NA
	17	1	619.00	597.55	911.10	979.45	1,204.96	NA	NA	NA	NA	NA	NA	NA	NA	NA
	18	1	415.40	1,684.78	1,381.06	1,390.68	1,492.20	NA	NA	NA	NA	NA	NA	NA	NA	NA
	19	2	211.69	411.30	519.20	480.05	587.85	636.53	851.99	861.89	1,077.76	NA	NA	NA	NA	NA
	20	1	2,258.51	1,509.49	1,449.94	2,449.75	NA	NA	NA	NA	NA	NA	NA	NA	NA	NA
	21	1	509.49	773.61	1,879.77	2,330.27	2,849.18	2,388.89	3,091.77	NA	NA	NA	NA	NA	NA	NA
	22	2	421.26	431.05	470.33	480.03	626.86	1,302.55	1,713.85	2,340.73	NA	NA	NA	NA	NA	NA
	23	2	219.49	411.25	450.46	793.40	744.26	802.95	910.80	1,234.03	1,429.01	NA	NA	NA	NA	NA
	24	1	493.98	421.25	715.02	1,048.06	NA	NA	NA	NA	NA	NA	NA	NA	NA	NA
	25	2	133.27	323.01	303.52	558.05	734.58	1,106.48	1,625.70	2,110.90	NA	NA	NA	NA	NA	NA
	26	1	1,261.53	1,997.23	1,958.32	1,351.10	3,017.99	NA	NA	NA	NA	NA	NA	NA	NA	NA
	27	2	141.00	616.99	518.81	714.67	1,096.64	1,224.15	1,498.32	NA	NA	NA	NA	NA	NA	NA
	28	2	125.48	293.68	205.73	284.14	264.45	538.65	675.81	587.73	989.41	918.61	NA	NA	NA	NA
	29	1	517.29	4,230.34	2,536.10	3,074.70	4,808.66	6,358.88	NA	NA	NA	NA	NA	NA	NA	NA
	30	2	148.86	146.87	88.18	117.35	137.09	205.64	254.65	382.07	NA	NA	NA	NA	NA	NA
	31	1	211.68	577.59	1,351.55	1,841.19	2,311.35	NA	NA	NA	NA	NA	NA	NA	NA	NA
	32	1	988.17	1,421.33	1,802.67	1,645.46	2,526.88	3,604.08	3,712.88	NA	NA	NA	NA	NA	NA	NA
	33	2	399.63	1,145.63	1,448.96	1,752.58	2,290.83	2,968.75	3,515.58	NA	NA	NA	NA	NA	NA	NA
	34	2	703.67	2,859.13	4,348.04	5,219.17	5,444.38	7,040.17	8,842.63	9,675.08	9,205.88	NA	NA	NA	NA	NA
	35	1	211.60	274.25	411.38	959.79	1,097.13	NA	NA	NA	NA	NA	NA	NA	NA	NA
	36	2	70.53	127.29	372.00	342.67	597.50	548.38	NA	NA	NA	NA	NA	NA	NA	NA
	37	2	148.80	293.58	215.46	401.63	519.00	803.04	577.92	754.21	822.88	NA	NA	NA	NA	NA
	38	1	814.77	2,614.46	1,194.75	1,762.92	3,006.33	2,898.80	NA	NA	NA	NA	NA	NA	NA	NA
	39	2	211.43	293.75	528.71	1,067.33	1,204.33	2,158.00	2,761.92	NA	NA	NA	NA	NA	NA	NA
	40	1	461.10	845.17	730.04	989.38	744.85	NA	NA	NA	NA	NA	NA	NA	NA	NA

		1=Women;	Power in W												
**Young**	Subject	2=Men	0	50	75	100	125	150	175	200	225	250	275	300	325	350
	1	2	101.92	137.13	235.13	146.92	146.90	264.48	225.33	391.68	332.93	450.42	773.80	812.70	587.55	1,052.95
	2	2	39.17	107.73	107.77	78.40	156.75	88.17	215.57	146.78	225.19	333.00	293.79	NA	NA	NA
	3	2	31.33	68.55	88.17	117.56	127.30	176.30	185.95	244.55	342.90	293.76	NA	NA	NA	NA
	4	2	54.86	107.83	166.60	176.37	303.75	303.74	352.74	489.93	NA	NA	NA	NA	NA	NA
	5	2	195.99	293.93	284.16	244.86	430.94	538.75	715.23	NA	NA	NA	NA	NA	NA	NA
	6	1	321.39	254.87	166.30	235.15	195.89	714.96	821.39	NA	NA	NA	NA	NA	NA	NA
	7	2	133.29	205.84	235.18	274.43	352.82	264.48	656.52	842.83	744.59	803.37	989.10	NA	NA	NA
	8	1	70.56	176.42	235.23	205.83	235.15	264.52	479.97	NA	NA	NA	NA	NA	NA	NA
	9	2	109.78	88.22	39.19	127.45	78.42	107.78	127.34	205.58	342.83	NA	NA	NA	NA	NA
	10	1	23.52	9.70	68.58	117.50	234.89	391.64	NA	NA	NA	NA	NA	NA	NA	NA
	11	2	54.90	117.53	185.96	127.36	274.23	97.97	254.65	352.62	479.71	597.53	646.52	1,175.40	971.43	NA
	12	2	94.05	29.40	137.20	97.96	137.13	117.40	176.34	166.53	254.68	254.67	333.11	715.01	695.54	749.31
	13	1	54.87	39.21	97.95	303.67	333.50	NA	NA	NA	NA	NA	NA	NA	NA	NA
	14	1	517.53	696.02	627.34	450.81	627.21	872.13	NA	NA	NA	NA	NA	NA	NA	NA
	15	1	462.67	362.60	323.47	333.16	313.48	509.38	489.57	822.95	1,165.80	NA	NA	NA	NA	NA
	16	2	360.51	744.61	1,332.98	626.98	744.68	489.93	470.32	636.77	1,126.96	1,127.13	1,538.28	1,413.94	NA	NA
	17	1	54.90	98.01	98.00	117.60	254.81	352.68	391.85	NA	NA	NA	NA	NA	NA	NA
	18	2	533.34	490.10	264.69	303.86	352.85	284.27	441.00	391.99	284.13	578.13	911.15	NA	NA	NA
	19	1	54.89	107.59	156.82	78.42	156.77	195.93	383.94	NA	NA	NA	NA	NA	NA	NA
	20	2	70.57	254.71	156.75	137.09	117.56	274.30	264.39	391.79	270.26	NA	NA	NA	NA	NA
	21	1	345.08	352.70	528.85	685.88	969.73	NA	NA	NA	NA	NA	NA	NA	NA	NA
	22	1	470.56	205.72	264.49	391.84	489.78	567.47	NA	NA	NA	NA	NA	NA	NA	NA
	23	2	86.26	186.22	88.19	88.18	127.40	176.33	244.87	342.90	509.23	546.09	NA	NA	NA	NA
	24	2	78.38	29.40	127.42	146.97	117.56	146.94	205.67	283.92	382.08	675.67	NA	NA	NA	NA
	25	2	54.91	58.82	68.50	49.01	68.54	117.56	78.38	156.78	137.16	205.73	293.88	338.24	NA	NA
	26	2	125.47	107.78	98.01	97.88	88.21	156.62	264.45	470.31	480.03	983.58	852.38	862.15	1,126.27	1,009.07
	27	1	70.58	98.00	127.43	127.30	196.00	98.03	323.20	NA	NA	NA	NA	NA	NA	NA
	28	2	164.61	205.83	342.92	146.92	254.78	323.41	450.80	215.61	284.17	411.41	NA	NA	NA	NA
	29	2	690.17	460.58	215.54	323.42	244.96	333.17	254.71	303.71	293.92	731.56	NA	NA	NA	NA
	30	1	250.93	431.33	323.42	352.88	333.21	548.67	676.19	NA	NA	NA	NA	NA	NA	NA
	31	1	125.46	244.99	215.59	274.34	313.38	313.50	470.12	842.46	NA	NA	NA	NA	NA	NA
	32	2	125.45	117.63	235.16	313.55	548.62	519.06	401.70	333.05	332.93	NA	NA	NA	NA	NA
	33	2	78.40	98.01	176.39	107.80	137.10	235.19	98.01	137.16	235.01	274.10	334.61	NA	NA	NA
	34	1	172.38	215.48	352.63	450.41	636.57	646.48	1,175.49	1,302.84	1,439.89	1,756.37	NA	NA	NA	NA
	35	1	30.73	105.67	192.00	662.29	1,555.42	2,353.25	NA	NA	NA	NA	NA	NA	NA	NA
	36	1	76.87	240.29	345.54	230.63	230.63	576.38	778.25	1,172.13	NA	NA	NA	NA	NA	NA
	37	1	84.63	48.08	115.42	115.21	307.54	432.42	NA	NA	NA	NA	NA	NA	NA	NA
	38	1	123.07	182.54	297.79	441.83	NA	NA	NA	NA	NA	NA	NA	NA	NA	NA
	39	1	107.77	124.88	96.08	249.96	269.00	333.38	NA	NA	NA	NA	NA	NA	NA	NA
	40	1	246.20	384.50	249.96	249.96	278.75	461.21	413.04	737.80	NA	NA	NA	NA	NA	NA

Aerosol particle emission in Particles/min

Old	Subject	1=Women; 2=Men	Power in W													
			0	25	50	75	100	125	150	175	200	225	250	275	300	325
	1	2	2,854.23	5,062.63	9,625.87	19,217.15	23,171.31	40,404.10	94,834.50	119,676.70	NA	NA	NA	NA	NA	NA
	2	2	12,000.55	30,361.55	39,071.65	45,891.07	104,512.07	155,092.76	385,773.71	NA	NA	NA	NA	NA	NA	NA
	3	1	4,846.26	17,682.80	31,986.75	52,884.08	60,182.98	NA	NA	NA	NA	NA	NA	NA	NA	NA
	4	2	3,052.32	12,174.66	18,002.12	23,965.58	52,660.35	81,338.67	106,986.92	NA	NA	NA	NA	NA	NA	NA
	5	2	3,994.02	11,419.86	15,048.73	16,672.85	26,037.05	21,772.14	39,148.98	38,972.56	49,173.23	49,454.73	NA	NA	NA	NA
	6	2	2,651.48	12,776.74	16,158.06	27,890.24	43,775.66	88,160.73	163,519.30	250,069.40	NA	NA	NA	NA	NA	NA
	7	1	4,974.67	20,971.86	39,952.44	95,990.46	238,754.28	472,904.28	NA	NA	NA	NA	NA	NA	NA	NA
	8	1	8,421.90	24,537.56	43,948.35	75,885.26	NA	NA	NA	NA	NA	NA	NA	NA	NA	NA
	9	1	2,413.24	10,239.01	15,523.92	58,180.41	162,423.56	189,582.24	NA	NA	NA	NA	NA	NA	NA	NA
	10	1	1,060.46	2,192.03	5,778.49	5,276.44	NA	NA	NA	NA	NA	NA	NA	NA	NA	NA
	11	1	1,633.30	47,165.87	62,991.78	122,276.69	129,940.40	NA	NA	NA	NA	NA	NA	NA	NA	NA
	12	2	2,299.84	2,908.65	3,730.62	3,500.82	12,188.48	24,942.47	38,199.03	47,116.06	NA	NA	NA	NA	NA	NA
	13	1	1,740.96	5,583.37	12,094.08	20,230.48	41,198.07	NA	NA	NA	NA	NA	NA	NA	NA	NA
	14	2	807.32	2,977.70	5,298.07	10,895.49	10,273.61	16,883.36	37,399.90	61,563.15	107,302.27	NA	NA	NA	NA	NA
	15	1	7,535.04	27,735.08	67,140.80	97,737.08	147,482.01	203,509.75	NA	NA	NA	NA	NA	NA	NA	NA
	16	2	2,195.10	7,704.22	15,488.27	27,618.73	39,295.33	78,718.46	152,512.77	239,957.17	328,533.20	NA	NA	NA	NA	NA
	17	1	5,859.71	12,973.89	33,738.08	52,374.45	73,069.98	NA	NA	NA	NA	NA	NA	NA	NA	NA
	18	1	3,886.58	28,195.29	30,352.22	58,881.91	88,307.95	NA	NA	NA	NA	NA	NA	NA	NA	NA
	19	2	3,267.62	10,645.23	18,662.71	21,546.56	28,278.64	39,764.64	61,762.98	75,199.18	113,545.14	NA	NA	NA	NA	NA
	20	1	22,442.14	12,427.53	32,816.23	68,338.49	NA	NA	NA	NA	NA	NA	NA	NA	NA	NA
	21	1	5,506.53	14,813.52	49,782.61	79,646.43	120,721.35	119,169.57	184,640.59	NA	NA	NA	NA	NA	NA	NA
	22	2	4,358.33	7,645.88	11,204.89	14,651.43	23,711.78	65,261.54	116,454.95	222,696.32	NA	NA	NA	NA	NA	NA
	23	2	2,441.75	11,735.93	17,246.58	36,780.87	39,596.05	50,447.41	67,686.71	114,511.61	154,637.84	NA	NA	NA	NA	NA
	24	1	3,438.48	6,201.70	17,794.19	42,466.98	NA	NA	NA	NA	NA	NA	NA	NA	NA	NA
	25	2	1,356.30	6,935.91	8,004.72	17,377.35	29,127.50	52,819.81	99,704.25	155,581.53	NA	NA	NA	NA	NA	NA
	26	1	11,700.20	36,273.98	52,145.97	25,878.03	142,357.42	NA	NA	NA	NA	NA	NA	NA	NA	NA
	27	2	1,841.16	14,013.38	14,829.45	26,450.09	51,781.03	76,993.12	119,154.61	NA	NA	NA	NA	NA	NA	NA
	28	2	1,372.64	5,267.39	4,691.36	7,954.69	9,984.96	24,882.54	37,516.91	39,426.50	72,875.47	67,945.17	NA	NA	NA	NA
	29	1	2,668.94	71,110.57	66,795.88	106,501.60	213,772.36	360,097.77	NA	NA	NA	NA	NA	NA	NA	NA
	30	2	1,545.62	2,920.51	2,277.82	3,901.06	5,554.10	10,284.88	15,451.38	28,257.31	NA	NA	NA	NA	NA	NA
	31	1	1,235.04	10,725.32	35,421.42	64,054.19	113,072.21	NA	NA	NA	NA	NA	NA	NA	NA	NA
	32	1	7,802.81	16,213.66	36,961.20	55,417.90	110,947.18	137,379.31	152,468.29	NA	NA	NA	NA	NA	NA	NA
	33	2	5,865.55	35,736.47	55,348.93	78,286.12	126,436.15	195,539.13	289,448.89	NA	NA	NA	NA	NA	NA	NA
	34	2	7,392.97	73,008.40	160,362.08	236,520.73	306,447.70	480,416.02	759,618.54	1,025,221.41	1,079,756.85	NA	NA	NA	NA	NA
	35	1	2,349.18	5,612.05	12,974.67	43,311.40	64,724.18	NA	NA	NA	NA	NA	NA	NA	NA	NA
	36	2	823.53	2,967.44	12,222.52	13,070.72	27,848.99	32,368.02	NA	NA	NA	NA	NA	NA	NA	NA
	37	2	1,054.82	7,121.67	6,557.69	15,874.17	24,756.13	45,280.30	41,209.53	64,469.14	87,573.02	NA	NA	NA	NA	NA
	38	1	6,417.84	46,719.70	34,227.65	70,556.91	168,113.40	181,017.06	NA	NA	NA	NA	NA	NA	NA	NA
	39	2	1,749.80	5,196.79	13,918.75	32,736.72	44,943.27	142,630.31	206,706.11	NA	NA	NA	NA	NA	NA	NA
	40	1	3,360.92	18,991.07	26,875.57	40,542.21	37,601.74	NA	NA	NA	NA	NA	NA	NA	NA	NA

Young	Subject	1=Women; 2=Men	Power in W													
			-	50.00	75.00	100.00	125.00	150.00	175.00	200.00	225.00	250.00	275.00	300.00	325.00	350.00
	1	2	1,721.32	3,594.52	8,076.48	6,015.79	7,110.97	14,197.78	13,575.19	26,786.99	25,609.12	38,745.81	75,038.61	86,459.21	71,826.29	141,811.97
	2	2	540.90	3,107.92	4,045.86	3,640.13	8,687.97	5,710.51	16,865.82	13,436.93	25,148.49	45,321.24	46,263.31	NA	NA	NA
	3	2	368.59	1,640.78	2,659.23	4,257.86	5,522.24	8,890.67	10,890.50	17,240.35	29,030.68	30,491.03	NA	NA	NA	NA
	4	2	392.33	2,177.24	4,356.82	6,136.35	12,972.06	16,987.28	25,871.54	43,243.95	NA	NA	NA	NA	NA	NA
	5	2	2,263.80	7,197.83	11,158.02	12,332.88	26,495.10	37,754.34	63,266.04	NA	NA	NA	NA	NA	NA	NA
	6	1	4,245.27	5,718.45	5,583.05	10,452.53	10,931.43	56,695.00	83,892.87	NA	NA	NA	NA	NA	NA	NA
	7	2	2,000.82	6,344.66	8,768.25	11,754.25	17,568.18	15,448.20	35,257.38	43,726.16	59,847.07	76,163.20	104,106.19	NA	NA	NA
	8	1	732.60	3,840.73	6,741.22	7,439.94	10,282.27	13,925.41	30,388.40	NA	NA	NA	NA	NA	NA	NA
	9	2	1,033.29	2,115.74	1,192.17	4,702.23	3,194.75	5,478.19	7,710.37	15,214.97	31,525.76	NA	NA	NA	NA	NA
	10	1	299.42	270.13	2,555.40	5,679.75	14,556.28	32,999.35	NA	NA	NA	NA	NA	NA	NA	NA
	11	2	872.97	3,784.31	7,369.95	5,839.59	14,352.89	5,794.69	15,919.30	24,005.97	41,321.80	58,919.03	77,349.21	170,256.59	144,391.00	NA
	12	2	1,375.73	734.06	3,988.18	3,575.40	6,115.94	5,993.59	9,714.83	10,935.76	19,028.99	21,928.07	32,403.53	80,519.37	90,413.13	101,208.34
	13	1	626.99	1,352.97	5,050.06	22,953.40	30,884.09	NA	NA	NA	NA	NA	NA	NA	NA	NA
	14	1	5,810.17	16,999.84	22,067.85	20,259.99	36,691.46	67,640.17	NA	NA	NA	NA	NA	NA	NA	NA
	15	1	3,153.80	7,855.79	9,918.08	12,065.51	12,944.43	26,466.60	29,018.87	62,137.40	114,937.43	NA	NA	NA	NA	NA
	16	2	5,269.89	18,840.50	45,258.49	25,529.69	33,381.94	26,000.91	28,682.72	42,658.84	79,704.56	85,364.35	135,042.42	168,124.81	NA	NA
	17	1	688.18	2,550.49	3,382.10	5,390.09	14,478.96	24,575.87	34,617.65	NA	NA	NA	NA	NA	NA	NA
	18	2	6,700.62	12,990.66	8,838.28	12,043.78	16,241.53	15,059.83	29,432.76	28,938.14	24,677.46	59,672.49	111,292.14	NA	NA	NA
	19	1	565.88	2,368.13	4,968.02	3,375.52	9,128.83	15,039.47	37,842.45	NA	NA	NA	NA	NA	NA	NA
	20	2	961.27	5,722.91	5,234.32	5,831.91	6,005.49	17,911.38	19,851.02	41,519.62	32,730.60	NA	NA	NA	NA	NA
	21	1	3,265.81	7,800.03	17,841.77	29,419.43	53,043.48	NA	NA	NA	NA	NA	NA	NA	NA	NA
	22	1	5,285.83	4,945.16	9,699.65	18,825.49	29,317.47	43,102.35	NA	NA	NA	NA	NA	NA	NA	NA
	23	2	1,127.42	4,283.60	2,656.23	3,275.68	5,754.67	9,424.30	16,090.67	29,192.03	55,523.36	73,582.49	NA	NA	NA	NA
	24	2	1,160.07	721.34	3,768.85	5,461.00	5,474.65	8,300.20	13,002.78	20,913.10	36,843.48	80,865.54	NA	NA	NA	NA
	25	2	935.68	1,703.79	2,481.30	2,139.96	3,385.64	6,785.38	5,168.04	11,819.32	11,963.14	19,239.40	38,290.76	50,515.34	NA	NA
	26	2	1,642.26	2,533.91	3,062.53	3,694.65	4,018.01	7,909.24	14,619.07	29,986.64	35,236.79	84,389.39	83,994.37	99,269.78	154,244.42	165,388.49
	27	1	565.52	1,760.63	3,547.05	4,375.72	8,623.61	5,265.04	22,211.56	NA	NA	NA	NA	NA	NA	NA
	28	2	2,853.87	4,955.43	12,109.25	6,296.14	13,026.65	19,335.29	31,495.14	20,772.73	31,357.66	54,241.41	NA	NA	NA	NA
	29	2	10,090.96	13,160.19	7,955.08	14,007.64	12,871.63	20,662.98	18,149.74	26,731.47	35,477.41	107,676.11	NA	NA	NA	NA
	30	1	2,097.99	8,511.72	9,864.95	13,815.05	17,262.32	37,086.93	55,301.88	NA	NA	NA	NA	NA	NA	NA
	31	1	1,084.49	5,306.25	6,347.32	10,111.36	13,701.89	16,599.36	31,658.66	71,721.48	NA	NA	NA	NA	NA	NA
	32	2	2,773.81	3,048.63	8,223.38	15,087.79	30,827.09	36,465.61	34,766.33	37,442.69	46,107.46	NA	NA	NA	NA	NA
	33	2	812.82	1,895.77	5,166.41	4,394.55	6,604.25	13,117.18	6,484.85	10,337.19	20,274.36	27,686.78	41,004.77	NA	NA	NA
	34	1	1,275.12	4,723.37	10,017.40	15,638.57	26,522.19	32,078.94	70,468.36	100,030.62	148,163.47	213,809.54	NA	NA	NA	NA
	35	1	270.75	2,152.43	5,307.37	24,497.74	71,621.77	150,220.51	NA	NA	NA	NA	NA	NA	NA	NA
	36	1	677.07	6,041.71	11,414.84	9,660.96	11,161.63	33,247.80	54,978.56	109,576.48	NA	NA	NA	NA	NA	NA
	37	1	607.96	1,217.84	4,163.65	5,525.58	19,442.26	35,391.57	NA	NA	NA	NA	NA	NA	NA	NA
	38	1	1,092.19	4,752.49	12,509.87	27,471.19	NA	NA	NA	NA	NA	NA	NA	NA	NA	NA
	39	1	613.42	2,435.66	2,813.94	9,490.45	14,709.22	27,109.71	NA	NA	NA	NA	NA	NA	NA	NA
	40	1	2,053.64	8,290.11	7,780.49	10,157.72	13,385.33	27,342.17	30,917.77	71,690.35	NA	NA	NA	NA	NA	NA

Aerosol particle emission in Particles/min

			percentage of exercise intensity					
			0	20	40	60	80	100
old	women	mean	13,223.55	20,253.40	31,712.00	50,902.91	84,003.87	142,896.80
		95% CI	4,268.35	6,078.85	9,042.06	14,483.10	25,805.37	51,266.82
	men	mean	9,211.96	16,872.75	31,278.17	58,720.28	111,722.81	215,593.59
		95% CI	6,306.17	11,456.56	20,877.68	38,169.08	70,096.68	129,680.94
young	women	mean	1,730.58	3,257.84	6,350.09	12,904.42	27,597.57	62,830.62
		95% CI	755.38	1,167.81	1,934.88	3,692.47	8,587.16	24,022.65
	men	mean	2,497.44	4,639.66	8,885.26	17,631.80	36,424.27	78,598.76
		95% CI	1,208.42	1,858.00	2,843.34	4,548.08	8,889.15	23,308.96

**Mean particle size per bin**			0.250	0.373	0.518	0.720	1.000	1.389	1.931	2.683	3.728	5.179	7.197
Rest	Young	Mean	4.01	0.77	0.26	0.10	0.04	0.02	0.00	0.00	0.00	0.00	0.00
		95%CI	0.43	0.36	0.26	0.19	0.14	0.10	0.07	0.05	0.04	0.03	0.02
	Old	Mean	3.20	1.12	0.61	0.34	0.12	0.02	0.00	0.00	0.00	0.00	0.00
		95%CI	0.36	0.30	0.22	0.16	0.11	0.08	0.06	0.04	0.03	0.02	0.02
Excercise	Young	Mean	2.83	1.53	0.81	0.31	0.10	0.02	0.00	0.00	0.00	0.00	0.00
		95%CI	0.29	0.25	0.18	0.13	0.09	0.07	0.05	0.03	0.02	0.02	0.01
	Old	Mean	1.70	1.41	1.24	0.65	0.20	0.03	0.01	0.00	0.00	0.00	0.00
		95%CI	0.28	0.24	0.17	0.12	0.09	0.06	0.05	0.03	0.02	0.02	0.01
